# Primary healthcare practitioners’ perspectives on trauma-informed primary care: a systematic review

**DOI:** 10.1186/s12875-024-02573-4

**Published:** 2024-09-12

**Authors:** Eleanor Bulford, Surriya Baloch, Jennifer Neil, Kelsey Hegarty

**Affiliations:** 1 Department of General Practice and Primary Care, University of Melbourne, Melbourne, VIC Australia; 2 Department of General Practice, Monash University, Melbourne, VIC Australia; 3 The Royal Women’s Hospital, Melbourne, VIC Australia

**Keywords:** Domestic violence, Trauma, Trauma-informed care, Primary healthcare, General practice

## Abstract

**Background:**

Exposure to domestic and family violence is a pervasive form of complex trauma and a major global public health problem. At the frontline of the health system, primary healthcare practitioners are uniquely placed to support individuals with experiences of trauma, yet their views on trauma-informed primary care are not well understood. This systematic review of qualitative literature sought to explore primary healthcare practitioners’ perspectives on trauma-informed primary care.

**Methods:**

Eight databases were searched up to July 2023. Studies were included if they consisted of empirical qualitative data, were conducted in general practice or equivalent generalist primary healthcare settings, and included the perspectives of primary healthcare practitioners where they could be distinguished from other participants in the analysis. Thematic synthesis was used for analysis.

**Results:**

13 papers met inclusion criteria, representing primary care settings from the United States, Canada, Australia, and Norway. Three key themes were developed: Changing the paradigm, Building trust, and Navigating the emotional load. Findings shed light on how primary healthcare practitioners perceive and strive to practise trauma-informed primary healthcare and the challenges of navigating complex, trauma-related work in the primary care environment.

**Conclusions:**

This review supports the need for recognition of the value of primary care in supporting patients with histories of trauma and violence, the development of interventions to mitigate the emotional load worn by primary healthcare practitioners, and further work to develop a deep and consistent understanding of what trauma-informed primary care encompasses.

## Introduction

Trauma is a public health issue of epidemic proportions [1]. In the 1990s, the landmark Adverse Childhood Experiences (ACEs) Study shed light on the strong associations between early life traumatic experiences and a wide range of mental and physical health outcomes, including depression, cardiac and respiratory disease, and cancers [2]; findings which are now supported by an increasingly large body of research [3–5]. One of the most pervasive forms of trauma is exposure to domestic violence, affecting approximately 1 in 3 women worldwide [6] and frequently constituting complex trauma, or trauma which is repetitive and cumulative [7]. Domestic violence is a leading cause of morbidity and mortality among women of child-bearing age, with significant health consequences including higher rates of depression, chronic pain, and harmful substance use [8]. Children, too, are profoundly affected by domestic violence within a family, with a range of impacts upon health both in childhood and later in life [9]. Particularly when exposure occurs in childhood, complex trauma such as domestic violence also frequently has neurodevelopmental impacts upon domains such as sense of self, somatic awareness, and emotional regulation [7].

For healthcare providers, trauma therefore is not only relevant as an important risk factor for mental and physical health outcomes, but may also have complex impacts upon the therapeutic relationship. While trauma and domestic violence do not discriminate, both historical and ongoing structural factors such as gender inequities, colonisation, racism, and poverty intersect to disproportionately affect specific populations, particularly women and First Nations communities [8, 10, 11]. The impacts of trauma can extend across generations, contributing to cycles of health inequity and social disadvantage [12, 13]. There is a critical urgency to optimise the response to trauma across healthcare systems.

The increasing recognition of trauma as a public health issue has led to the conceptualisation of trauma-informed approaches to healthcare [14]. The Substance Abuse and Mental Health Services Administration (SAMHSA) outlines four key assumptions for trauma-informed services: a basic realisation of trauma and its impacts; a recognition of the signs of trauma; responding through applying trauma-informed principles across all areas of service delivery; and resisting re-traumatisation of both clients and staff members through providing a safe environment [15]. Key principles of trauma-informed care include safety; trustworthiness and transparency; peer support; collaboration and mutuality; empowerment, voice, and choice; and cultural, historical, and gender issues [15]. A strengths-based approach, trauma-informed healthcare aims to build patients’ sense of resilience and of agency, to reduce the risk of causing patients harm, and to offer opportunities for healing [14, 16, 17].

As the frontline of the healthcare system, primary healthcare practitioners are likely to be regularly seeing and managing the health consequences of trauma [17–19]. General practitioners (GPs) are one of the most common groups that women tell about their experiences of domestic violence [20] and primary care has been highlighted internationally as a priority setting for the healthcare response to domestic violence [8]. However, primary healthcare practitioners may face a range of systemic and personal barriers to identifying and addressing their patients’ experiences of abuse [21, 22]. Primary healthcare services that do not adequately recognise or understand trauma not only miss opportunities to optimise health outcomes for their patients, but also risk causing further trauma [15, 17].

Nonetheless, primary healthcare environments such as general practice are in many ways uniquely positioned to support individuals with experiences of trauma and violence. The generalist lens, community setting, and potential for long-term therapeutic relationships and continuity of care arguably align well with the key principles of trauma-informed care [23–26]. Evidence has been building to support the value of trauma- and violence-informed primary healthcare for First Nations women [10] and limited studies of trauma-informed training interventions in primary healthcare have shown promise [27–29]. However, the practicalities of how the principles of trauma-informed care should be implemented in primary healthcare remain an area of limited evidence [30, 31]. In order to understand how trauma-informed approaches to healthcare can be put into practice, understanding the perspectives and first-hand experiences of frontline practitioners is essential. There is currently very little insight into how primary healthcare practitioners understand and view trauma-informed primary healthcare, with no previous systematic reviews focusing on this. To help address this gap, we conducted a systematic review of qualitative literature exploring primary healthcare practitioners’ perspectives on trauma-informed care.

## Methods

### Search strategy

Eight databases were searched in July 2023: ASSIA, MEDLINE, CINAHL, Embase, Global Health, PsycINFO, SocINDEX, and Web of Science. The MEDLINE search strategy was initially designed using subject headings and keywords for trauma-informed care and primary healthcare and was subsequently translated to fit other databases. The MEDLINE search strategy is outlined in Table 1.

**Table 1:** MEDLINE search strategy

#	Searches
1	(trauma adj3 informed).mp.
2	(general practic* or gp or family doctor* or primary care).mp.
3	trauma informed care.mp.
4	trauma sensitive care.mp.
5	(Health adj3 Care*).mp.
6	(Primary adj3 care).mp.
7	(Basic adj3 health).mp.
8	2 or 5 or 6 or 7
9	1 or 3 or 4
10	8 and 9

### Inclusion criteria

We included empirical studies that used qualitative methodology and analysis, were conducted in general practice or equivalent generalist primary healthcare settings, and included the perspectives of primary healthcare practitioners. We chose to include qualitative research only, to allow an in-depth analysis of practitioners’ perspectives and experiences. Studies that included other groups, such as patients, were included only if primary healthcare practitioners could be separated from other participants in the analysis. If the perspectives of healthcare practitioners from other disciplines were explored, a paper was included if at least 50% of healthcare practitioner participants were from primary care settings. We only included studies published in English and did not apply a date range limit. Studies were excluded if they were conducted in non-generalist primary healthcare settings, were non-empirical papers such as commentaries or reviews, if they did not include the perspectives of primary healthcare practitioners, or if primary healthcare practitioners were unable to be distinguished from other participants in the analysis.

### Selection of studies

**Fig. 1:**
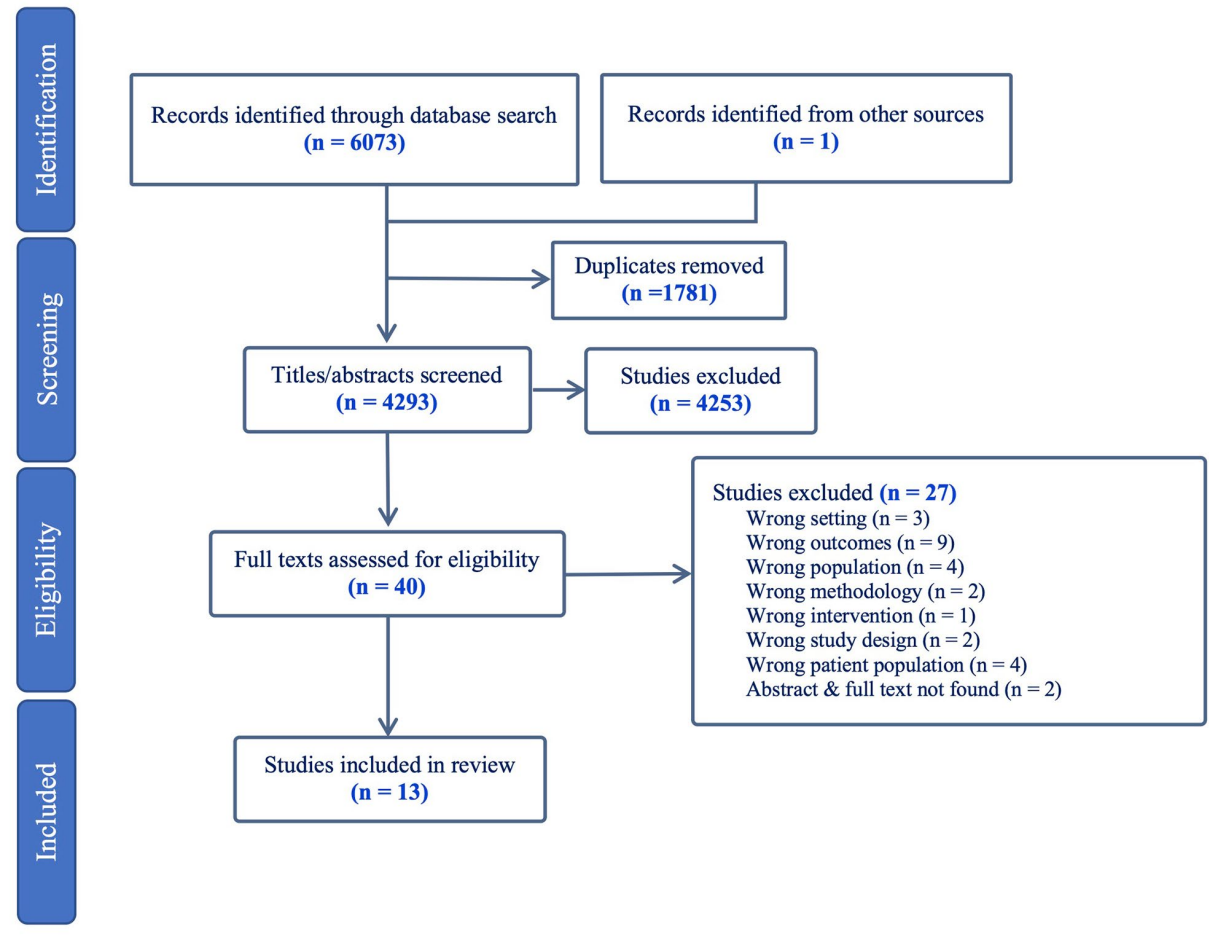
Flowchart of the study selection

Two reviewers (EB and SB, and subsequently EB and JN) independently screened each title and abstract against inclusion criteria using the software program Covidence [32]. Following initial screening, full-text papers were independently reviewed and identified for inclusion or exclusion. Any disagreements about study inclusion were resolved through discussion with a third reviewer (KH). The flow of studies is displayed in Fig. 1.

### Data extraction and synthesis

EB and SB extracted data into a standardised form, including details on study date, country, aims, methodology, setting, and sample size and characteristics. We imported results data into the qualitative data analysis software NVivo [33]. EB conducted a thematic synthesis as guided by the methodology of Thomas and Harden [34], in consultation with SB, JN, and KH. Primary data from each results section, including participant quotes and authors’ interpretations, were read and re-read, assessed on a line-by-line basis, and coded. Codes were organised into descriptive themes and finally, developed into analytical themes based on further careful examination and analysis through the lens of the research question.

### Quality appraisal

EB used the Critical Appraisal Skills Programme (CASP) tool to appraise the quality of each included study [35]. The tool assesses each study’s aim, research design, recruitment, data collection, reflexivity, ethics, data analysis, statement of findings, and overall value. For each domain, it was assessed whether each paper fully, partially, or did not meet expected standards or if it was unclear.

### Review author reflexivity

The review team consisted of researchers working in domestic and family violence and primary healthcare research. All authors are also medical practitioners with clinical experience in general practice and women’s health. At the outset of the review, all authors believed in the importance of trauma-informed approaches to care and in the valuable role that primary healthcare can play in the response to trauma and abuse. The authors’ views and experiences may influence the analysis of this review.

## Results

### Overview of studies

We identified 13 studies (Table 2) that met inclusion criteria, published between 2011 and 2023. Studies were conducted in the United States (seven studies), Canada (three studies), Australia (two studies), and Norway (one study). Settings included a veteran affairs primary care clinic [36]; a general practice clinic for young women in a socially disadvantaged community [37]; First Nations health services in Australia [38], Canada [39], and the United States [40]; and both urban and rural generalist primary healthcare clinics, five of which were described as serving low socio-economic or marginalised communities [41–45]. Data was collected via both individual interviews (nine studies) and focus groups (seven studies), with several studies using both methods. One study used yarning interview methodology [38]. Participants included doctors, nurses, social workers, and non-clinical staff working in primary healthcare. All studies used qualitative data analysis methodology, with most using thematic analysis. The overall quality of papers was high, although many papers did not discuss their consideration of the relationship between researchers and participants (Table 3).

**Table 2:** Study and participant characteristics

Author and year	Country and setting	Primary research aim	Qualitative method (data analysis)	Primary healthcare practitioner sample
Bergman et al 2019 [36]	United States Veterans’ Health Administration primary care clinics	To better understand ways in which Veterans Health Administration primary care providers in mixed-gender primary care clinics currently deliver primary care to women veterans with sexual trauma histories, such as military sexual trauma, to inform strategies for improving trauma-sensitive primary care	Interviews (thematic analysis)	*N* = 28 (21 doctors, 7 nurse practitioners)
Brooks et al. 2018 [37]	Australia Young women’s clinic for women aged 12–25 years	To explore how trauma-informed care can support recovery from adversity and illness	Focus groups and interviews (thematic analysis)	*N* = 12 (2 general practitioners, 2 nurses, 2 receptionists, 1 social work student, 1 art therapist, 1 former manager)
Browne et al. 2018 [41]	Canada Urban and rural primary care clinics	To illustrate the process and impacts of implementing an organizational-level health equity intervention aimed at enhancing capacity to provide equity-oriented health care	Interviews (thematic analysis)	*N* = 31 (5 registered nurses, 5 nurse practitioners, 3 physicians, 4 clinic leaders, 5 social service providers, 3 medical office assistants, 3 administrative staff, 3 staff in other roles)
Browne et al. 2016 [39]	Canada Urban Aboriginal health centres	To offer a framework and specific strategies for promoting equity-oriented care that takes into account the colonial history and ongoing subjugation of Indigenous peoples, and that supports Indigenous peoples’ agency and resistance to such subjugation	Interviews and focus groups (interpretive thematic analysis)	*N* = 41 (10 nurses/nurse practitioners, 9 physicians, 9 medical office assistants and office managers, 4 administration, 3 social workers, 2 substance use counsellors, 4 other staff)
Castellanos et al. 2023 [44]	United States Safety net primary care clinics in an urban area	To describe how intersecting interpersonal and structural trauma inform the personal and clinical care trajectories of patients with chronic non-cancer pain who experienced reductions in or discontinuation of opioid prescriptions in safety-net primary care settings	Interviews (modified grounded theory analysis)	*N* = 23 (physicians, nurse practitioners, and physician assistants providing longitudinal primary care – breakdown not specified)
Chung et al. 2012 [42]	United States Safety net primary care clinics from urban and rural areas	To describe the barriers and facilitators of appropriate mental healthcare for trauma-related mental health problems among low-income primary care patients from the perspectives of clinicians and administrators	Interview and focus groups (thematic analysis)	*N* = 32 (10 primary care administrators, 9 physicians, 8 nurses, 5 medical directors, 4 executive/clinical director/admin)
Cullen et al. 2020 [38]	Australia Aboriginal Community Controlled Health Organisations	A case study inquiry into how Aboriginal Community Controlled Health Organisations integrate trauma- and violence-informed care through systemic decolonisation	Yarning interviews (collaborative framework and thematic analysis)	*N* = 24 (11 management/leadership staff, 13 clinical and allied health including GPs, Aboriginal health practitioners, nurses, midwives, mental health nurses)
Green et al. 2011 [46]	United States Primary healthcare services	To increase knowledge about provider perspectives on provision of health care to trauma patients in primary care settings for underserved clients and to inform the development of effective interventions for patients and providers	Focus groups (iterative coding)	*N* = 31 (including family medicine residents, internal medicine practitioners, nurse practitioners – breakdown not specified)
Hiratsuka et al. 2017 [40]	United States First Nations primary healthcare services	To determine considerations and recommendations provided by patients, health care providers, health care administrators, and tribal leaders in the development of an adult trauma screening, brief intervention, and referral for treatment process to pilot at two large First Nations primary care systems	Interviews and focus groups (thematic network approach)	*N* = 24 (clinical/administrative/tribal leaders and healthcare providers – breakdown not specified)
Levine et al. 2021 [43]	Canada Primary care clinics	To explore the perspectives of primary healthcare staff on the impacts of interprofessional trauma- and violence-informed care education	Interviews (thematic analysis)	*N* = 14 (from medicine, nursing, pharmacy, social work, counselling, administration and leadership – breakdown not specified)
Matthew et al. 2022 [45]	United States Academic urban primary care clinic	To solicit perspectives and opinions grounded in patients’ and providers’/staff lived experience within a trauma-informed care model in an ambulatory healthcare setting serving women and children	Focus groups (thematic analysis)	*N* = 30 (17 nurses/medical assistants, 4 physicians or nurse practitioners, 3 mental/behavioural health staff, 2 administrative/leadership, 4 other)
Rønneberg et al. 2022 [48]	Norway General practice	To understand GPs’ perceptions of the medical relevance of patients’ stories of painful and adverse life experiences and what hinders or facilitates working with such stories	Focus groups (reflective thematic analysis)	*N* = 18 (general practitioners)
Van Den Berk-Clark 2021 [47]	United States Urban and suburban primary healthcare practices	To identify barriers to screening and to determine strategies for implementing trauma and post-traumatic stress disorder screening and treatment in primary care settings	Interviews (content analyses)	*N* = 10 (primary care physicians)

**Table 3:** Critical Appraisal Skills Programme (CASP) checklist [35]

	Bergman et al. (2018)	Brooks et al. (2018)	Browne et al. (2016)	Browne et al. (2018)	Castellanos et al. (2023)	Chung et al. (2012)	Cullen et al. (2020)	Green et al. (2011)	Hiratsuka et al. (2017)	Levine et al. (2021)	Matthew et al. (2022)	Rønneberg et al. (2022)	Van den Berk-Clark et al. (2021)
Clear statement of the aims of the research?	Y	Y	Y	Y	Y	Y	Y	Y	Y	Y	Y	Y	Y
Qualitative methodology appropriate?	Y	Y	Y	Y	Y	Y	Y	Y	Y	Y	Y	Y	Y
Research design appropriate to address the aims of the research?	Y	Y	Y	Y	Y	Y	Y	Y	Y	Y	Y	Y	Y
Recruitment strategy appropriate?	Y	C	C	Y	Y	C	Y	C	Y	Y	Y	Y	C
Data collected in a way that addressed the research issue?	Y	Y	Y	Y	Y	Y	Y	Y	Y	Y	Y	Y	Y
Relationship between researcher and participants adequately considered?	C	C	C	C	C	C	*P*	C	C	C	*P*	Y	*P*
Ethical issues taken into consideration?	C	Y	*P*	C	C	C	*P*	C	C	Y	Y	Y	Y
Data analysis sufficiently rigorous?	Y	C	Y	Y	Y	C	Y	Y	Y	Y	Y	Y	Y
Clear statement of findings?	Y	Y	Y	Y	Y	Y	Y	Y	Y	Y	Y	Y	Y

Y = yes

N = no

*P* = partial

C = can’t tell

## Key themes

Three key themes were developed that described primary healthcare practitioners’ perspectives on trauma-informed care: *Changing the paradigm*, *Building trust* and *Navigating the emotional load.* The key themes and sub-themes are discussed in detail as follows.

### Changing the paradigm: “I’m at the same level as they are”

A key theme identified across 10 of the 13 studies was the ways in which trauma-informed care could encompass shifts in how primary care practitioners saw the meaning, purpose, and framework of their work [36–43, 46, 47]. This theme is explored across three subthemes: shifting the biomedical lens, recognising and understanding trauma, and being an advocate.

#### Shifting the biomedical lens

Primary healthcare practitioners described how trauma-informed care involved a shift in the traditional biomedical lens, towards a more holistic viewpoint that encompassed a strong awareness of the impacts of trauma and of the broader psychosocial, spiritual, societal, and historical influences on their patients’ health [39, 43]. Primary care practitioners in several studies discussed the importance of acknowledging and challenging power imbalances, including both within the clinical relationship and between healthcare disciplines [38, 39, 41]. One practitioner at a Canadian Aboriginal health centre said:

*“I want to make this person feel that even though I’m a [provider]*, *I am at the same level as they are. I don’t place myself above them or anything like that. There is no status of power when I work with people. I try to keep that as minimal as possible.”* [39].

In some cases, this led to changes in dynamics among the interdisciplinary staff working within the clinic. In a Canadian study on the impacts of an equity-oriented primary healthcare intervention, which included a significant trauma- and violence-informed care component, one staff member observed:

*“In the meetings*, *it’s starting to shift*, *which is really big*, *because for years*, *we’ve been saying*, *ok*, *we need the psychosocial piece to come out in the meetings and not talk three quarters of the time about the medical stuff.”* [41].

In First Nations health settings, staff discussed the fundamental importance of recognising colonisation and racism as major ongoing structural sources of trauma and violence impacting upon their patients’ health [38, 39]. In contrast to other included studies, however, one study of Norwegian GPs’ perceptions of the medical relevance of their patients’ adverse life experiences [48] found some different perspectives. While some GPs were confident in taking a holistic approach that acknowledged their patients’ experiences of trauma, others were sceptical of connections between trauma and health and maintained more of a traditional biomedical perspective in their role:

*Some GPs in all three focus groups expressed uncertainty as to whether work with painful and adverse experiences fits into the scope or mandate of a busy GP’s clinical practice*, *irrespective of the stories’ potential medical relevance.* [48]

#### Recognising and understanding trauma

Most studies explored the ways in which primary care practitioners picked up on patient indicators that may suggest an underlying history of trauma. These included both clinical presentations such as chronic pain [39, 44, 47] and non-specific somatic symptoms [42, 43, 47, 48], but also patient behaviours and clinical interactions [36, 37, 46]:

*“I had... a prenatal patient and I was doing an... initial pelvic exam on her*, *she just started breaking down in tears... when I’m putting a speculum in... and then I asked her*, *because that totally went off in my head*, *‘Okay*, *there’s something really off here.’”* [46].

For some practitioners, recognition of underlying trauma shifted their perspective on their patients’ care, particularly for so-called “difficult” patients:

*“If the primary provider can recognize that this poor difficult patient is in fact a person in pain with huge problems and will need quite a bit of attention... It’s much more complicated than just heart failure or diabetes. This is what they call pain heart. Pain hearts need a lot of attention; a lot of love.”* [36].

#### Being an advocate

Primary care practitioners described their role in providing trauma-informed care as extending beyond the provision of immediate medical care to being an advocate for their patients more broadly [37, 38, 43]. Practitioners discussed how they supported patients to access housing, social security, employment, and education and their role in helping patients to navigate complex systems [37, 38]. Using their positions to advocate at broader systems and societal levels was also seen as important [38, 43]. In one study at an Australian clinic for young women, the authors stated:

*Staff saw advocacy as another crucial aspect of trauma-informed care. GPs often wrote letters or made phone calls on behalf of clients*, *pressing for affordable housing*, *affordable care with medical specialists*, *as well as advocacy with Centrelink*, *employment agencies*, *schools*, *university and TAFE (Technical and Further Education institutions).* [37]

In Levine and colleagues’ interviews with primary healthcare staff about the impact of interprofessional education on trauma- and violence-informed care, one doctor described how the framework had given them greater confidence to advocate around broader structural issues affecting their patients:

*“It made me sort of feel confident enough to start doing something about [trauma and violence] when I see it. So we had a discussion today about*, *in a sharing circle*, *about First Nations people and their interactions with police.”* [43].

Practitioners spoke about involving other practitioners and services in the care of patients with histories of trauma, particularly mental health services [36, 42, 46, 47]. Multiple problems with referral pathways were discussed, including not enough services [36, 40, 44, 47], long wait times [40], the potential for fragmentation of care [36], and other providers providing services in a manner that was not trauma-sensitive [38, 39]. Having a strong knowledge of local services; strong partnerships and communication between services; and being prepared to advocate for patients to be seen in a timely manner were all explored as important facilitators [36, 38, 42, 47].

### Building trust: “Just moving one step at a time”

The central importance of building a strong, trusting relationship with patients was highlighted across seven studies [36, 37, 40, 45–48]. Allowing appropriate time and space, sensitively approaching physical examinations, and communicating effectively were sub-themes that illustrated the process of building trust with patients.

#### Allowing time and space

Primary care practitioners in several studies described how building trusting relationships with their patients required moving away from a need to solve their patients’ problems towards being prepared to meet patients where they were at, and sometimes to simply listen [37, 40, 46, 48]. This required allowing time and space to work with patients at their own pace:

*“Establish rapport with the patient. Some sense of relationship. Trust in that provider. I guess seeking just what the patient is willing to acknowledge they need help with… just moving one step at a time.”* [40].

One GP at the Australian young women’s clinic described the concept of “holding”:

*“As a doctor you want to be able to fix someone or solve their problems and then move on*, *but a lot of the time with Young Women’s Clinic it is about keeping them safe*, *reduction of harm*, *minimising other outside impacts on their lives until they get to a stage where they are able to move on. So that holding is a very important part of it…”* [37].

In Rønneberg and colleagues’ study with Norwegian GPs, however, several participants expressed a different view that was less aligned with the concept of allowing time and space:

*“[Working with stories of painful and adverse experiences] doesn’t fit with my daily routines as a GP. Problems have to be solved then and there.”* [48].

While allowing adequate time for consultations was considered important by many, practitioners stressed the challenges of time pressures in the primary care environment [36, 43, 45, 47, 48]. These included insufficient time to manage the complexity of trauma-related issues, to provide appropriate counselling and support, to carefully navigate sensitive physical examinations, and for practitioners to have the opportunity to critically reflect on their own practice:

*“The [clinic] time I needed*, *I didn’t get. They [women veterans who screen positive for a history of sexual trauma] really need counselling and we are the first line of people as primary care. It’s so easy to lose them after the first visit.”* [36].

*“You cannot help them in a 15-minute appointment”* [45].

#### Navigating physical examinations

Conducting sensitive and respectful physical examinations was highlighted in several studies as an important, yet at times challenging aspect of providing trauma-informed primary care and building trust with patients [36, 37, 46]. Intimate examinations such as pelvic and breast examinations and procedures such as cervical screening tests were highlighted as an especially complex area to approach, particularly with patients who had experienced sexual trauma [36, 37, 46]. Practitioners described the importance of allowing appropriate time to prepare for and conduct physical examinations, but were often faced with uncertainty about the most appropriate way to proceed.

A staff member in the Australian young women’s clinic recounted:

*“I remember one woman where the nurse met with her for six months*, *just talking about Pap smears... She had a very strong child sexual assault history and I don’t think she’d ever had a Pap smear*, *and she did finally get there.”* [37].

#### Being mindful of communication

Practitioners in several studies described being careful in their choice of language when discussing trauma-related issues with their patients [36, 46], and being aware of their verbal and non-verbal signals and the potential for these to evoke a patient’s trauma [40, 41, 43, 48]. Listening in a non-judgemental way, being patient, and providing validation were all highlighted as important parts of communicating with patients in a trauma-informed manner [36, 39, 40, 48].

Reflecting on the impact of interprofessional trauma- and violence-informed care education, one clinician said:

*“Before it was like*, *I know I’ve got to get all this stuff done before I go... but then I realize I’m going well wait a minute*, *if I’m just spewing something back or I’m not making eye contact*, *you know*, *I’m causing that person trauma”* [43].

Another practitioner in the veterans’ healthcare setting described the need to be careful with their communication:

*“It’s a challenge to take care of her [patient] because…she cancels appointments. So*, *sometimes I can be irritated or whatever. She’s very*, *very sensitive to my verbal cues. And so*, *I’ve got to really tone it down.”* [36].

Two studies touched on how discussing the links between adverse life experiences and health with patients could at times lead to complex and challenging conversations, particularly when resources to help address underlying trauma and social determinants were limited [44, 48]:

*“People are very focused on kind of physical causes or a specific thing. And it’s a hard conversation to have about how that pain arises...and they often see that as [...] “you just don’t want me to take these medicines [opioids] that would help me””* [44].

### Navigating the emotional load: “Your brain is working, your heart is working”

Ten papers described the emotional load that primary care practitioners carried in their work, including experiences of secondary trauma [37, 39, 41, 43, 45] and feelings of uncertainty and being overwhelmed [36, 42–44, 46, 48]. A small number of studies highlighted sources of personal support that some practitioners drew upon [43, 45].

#### Secondary trauma

Experiences of secondary or vicarious trauma among primary care practitioners arose in several studies [37, 39, 41, 45]. These experiences were described as leading to staff distress [37], compassion fatigue [41], burnout [41], exhaustion [45], and challenges with staff retention [39].

As one practitioner, who was working at an urban primary care clinic in a low socioeconomic community, described:

*“It’s wearing. It’s hard. You can’t keep up. It’s emotionally and physically draining. Your brain is working*, *your heart is working*, *you’re physically working.”* [45].

#### Feeling uncertain and overwhelmed

Primary care practitioners described how the complexity of navigating trauma-related issues in primary care could leave them feeling apprehensive, anxious, and uncertain of how best to proceed [36, 43, 46, 48]. Particular areas of uncertainty included how to approach conversations related to trauma [36, 46, 48], conducting examinations on patients with a trauma history [36], and navigating a tension between addressing a patient’s immediate needs and the underlying experiences of trauma or violence perpetuating their medical issues [43, 44]. At times practitioners doubted their own abilities [36, 46, 48], could feel overwhelmed by the complexity of the work [42, 46], and were frustrated at a perceived powerlessness to help their patients [41, 44–46].

*“When you’re dealing with trauma sometimes you wonder how much you should be doing at any particular moment. Somebody comes in because their ankle is sprained and they sprained their ankle fleeing an abusive partner... do you just... help the person with the ankle because that’s what they came in for or do you try to remove them from a violent situation?”* [43].

*“I feel more apprehensive overall…basically unsure and kind of nervous when I do take care of a patient who has had a history of military sexual trauma.”* [36].

*“Oh God*, *there’s really nothing you can do for them*, *you just want to leave*, *then you can’t do that*, *that’s not right*, *so you’ve got to do something.”* [46].

#### Sources of personal support

Despite the challenges of a heavy emotional load, two studies found a sense of personal meaning that supported primary care practitioners in their ability to provide trauma-informed primary care. Practitioners in one study described how the framework of trauma-informed care aligned to their personal values and sense of vocation [43], while others described feelings of fulfilment [45] and the importance of support from colleagues and peers [43, 45]:

*“We have a work environment that allows us to be supportive of each other so that we can have the emotional reserves to be able to provide trauma informed care. I think we’re flexible: if you have somebody that comes in that’s in a particular trauma or crisis*, *our colleagues are always really good about accommodating that time.”* [43].

*“I think when I can finish a day having helped somebody... that’s what keeps me going.”* [45].

## Discussion

This review of 13 qualitative studies sheds light on primary care practitioners’ perspectives on trauma-informed care in general practice and equivalent primary healthcare settings. The three main themes developed were: Changing the paradigm, Building trust, and Navigating the emotional load. These concepts have been discussed in the trauma literature to some extent but have not previously been synthesised from the voices of primary healthcare practitioners [10, 30, 49, 50].

Primary care practitioners in the included studies saw trauma-informed care as requiring a shift from a typical biomedical viewpoint towards a more holistic lens, which recognised indicators of trauma in their patients’ presentations and considered the impacts of structural factors and power dynamics. Taking a trauma-informed approach also meant the practitioner’s role broadening to include a strong advocacy component, supporting their patients to navigate complex systems and using their role to help patients to access basic needs such as housing and social services. This demonstrated a recognition of the impact of ongoing social determinants on the health of patients with experiences of trauma and the practitioner’s ability to support their patients as an ally as much as a clinician. Being an ally and providing patient-centred, practical support have consistently been emphasised by women with experiences of domestic and sexual violence as one of the most important things they seek from healthcare professionals [50, 51]. For the clinician themselves, adopting an advocacy lens is also known to be a key supportive factor in addressing domestic violence and abuse [21].

Primary care practitioners across the studies in this review emphasised the importance of building trust with their patients, highlighting both the unique challenges and opportunities that the general practice setting provided. Developing trust has been highlighted repeatedly as an essential element to caring for people affected by trauma and violence [10, 21, 49, 51, 52], recognising that individuals may have prior experiences of betrayal of trust and of disrupted attachment. Investing the time to build a strong clinical relationship, taking a non-judgemental approach, and being prepared to sit with patients where they were at all supported practitioners in their provision of trauma-informed care. This again reflected moving away from the traditional medical mindset of solving a patient’s problems and reflected the value of the long-term care that is frequently possible in primary care settings. Accepting this shift away from the need to be a ‘fixer’ has previously been found to support practitioners to feel more confident and ready to address domestic violence [21].

The concept of “holding” in general practice has been described in limited literature [37, 53] and refers to the development of trusting patient-clinician relationships, general practice as a ‘safe space’, and ongoing empathic support and advocacy without expectation of cure. Recent research around responding to child abuse and neglect in primary care settings has described how clinicians use holding strategies to navigate a ‘grey zone’ of clinical uncertainty; striving to create emotionally safe spaces, build an ongoing therapeutic relationship, and implement practical strategies to address families’ vulnerability [54]. While ‘holding’ may be simply putting a name to an approach that many primary care clinicians have instinctively understood for years [53], there is room to build our understanding of this concept and to recognise the unique value that primary care adds to the care of patients with complex trauma-related health issues. A small number of studies in this review did, however, report on feelings of frustration experienced by some clinicians when they felt unable to quickly solve their patients’ trauma-related issues. There may be varying degrees of comfort across the profession with taking a ‘holding’ approach and as such, strategies to build primary care practitioners’ capacity and confidence with the role they can play within such ‘grey zones’ may be of value.

The paradigm shift described across the studies in this review partly mirrors the growing understanding of generalism as a skillset in its own right [25]. In their exploration of the craft of generalism, Lynch and colleagues (2022) describe four key principles: whole person scope, relational process, healing orientation, and integrative wisdom [25]. The practice of trauma-informed primary healthcare as explored through the first-hand experiences and perspectives of practitioners in this review indicates a close alignment between generalist and trauma-informed philosophies, supporting the unique value of generalist healthcare such as general practice in supporting patients with experiences of trauma and violence.

Consistent with other literature around managing complex issues in general practice [22, 55, 56], time constraints were highlighted as a key challenge that practitioners were constantly navigating as they strove to build a trusting clinical relationship with their patients. Conducting physical examinations in a trauma-informed way was also an important challenge for primary care clinicians, reflecting the unique nature of a generalist speciality where both mental and physical healthcare must be sensitively navigated. While trauma-informed physical examination is starting to be integrated into some medical education curricula [57], literature exploring trauma-informed physical examinations for primary care professionals remains limited at present.

This review also highlighted the emotional load that primary care practitioners were navigating. Many of the practitioners interviewed across the included studies were working in marginalised communities with high rates of trauma. Being prepared to allow time and space and take a slow approach to building trust, while managing challenging trauma-related health issues, meant practitioners were often carrying a heavy burden of uncertainty and complexity. Several studies reported on experiences of secondary trauma and of feeling overwhelmed. Time, resourcing, and other systems-level barriers continued to add additional pressures to already complex work. The emotional labour of navigating trauma-related issues in general practice, and how this can lead to burnout and vicarious trauma, has been described previously in limited literature [58, 59], but the evidence base for how to best support primary care practitioner wellbeing is currently minimal [60]. A small number of studies in this review, however, reported on a personal sense of meaning that helped to support practitioners in their work. This is again consistent with systematic review data which indicates that having a personal commitment to addressing domestic and family violence, such as through a feminist or human rights lens, is a strong supporting factor for health practitioners who feel prepared to engage with trauma and violence-related issues [21].

### Strengths and limitations

To our knowledge, this is the first systematic review of qualitative studies exploring primary care practitioners’ perspectives on trauma-informed care. A robust search strategy yielded a large number of papers that were independently screened by two reviewers. Several limitations may apply. The number of papers that met inclusion criteria is small. Studies explored trauma-informed care from slightly different angles, including in the context of specific interventions, which may impact the application of the findings to other settings.

A number of different primary healthcare settings were represented, including urban, rural, and First Nations health services, as were the perspectives of primary healthcare staff from several different professional disciplines (including medicine, nursing, social work, and administration). However, all studies were from high income, Western countries. This is likely to also limit the transferability of the findings. While this review was focused on healthcare professional perspectives, the voices of consumers and community members, including those from diverse backgrounds and with different and intersecting life experiences must be central to the ongoing conceptualisation of trauma-informed primary care.

While the contemporary understanding of the complex impacts of trauma and violence is ever evolving, there remains a lack of robust evidence around trauma-informed interventions in primary care [30]. In this review, while consistent themes were able to be identified across the small number of included studies, it is not possible to be certain that each study reflects a shared understanding of trauma-informed care across primary healthcare. Furthermore, while this review focused on trauma-informed care, we recognise and also support the broader concept of trauma- and violence-informed care, which builds on trauma-informed care to add a stronger focus on the intersection of structural inequities and systemic violence with interpersonal violence [10, 39, 61]. Ongoing research that further defines what trauma- and violence-informed healthcare looks like and builds an evidence base for the primary care setting is needed.

### Implications and conclusion

The conceptualisation of trauma-informed care as a paradigm shift inevitably requires all levels of the healthcare system to understand and adopt this approach. While primary care practitioners discussed many ways in which they built trauma-informed principles into their own practice, several systems-level factors such as time structures, resourcing, and referral pathways did not consistently support them in undertaking this work [21, 26]. Working towards a shared understanding of trauma-informed principles and integration of these across all services and systems is essential and requires ongoing focus. Funding structures must recognise and reflect the value of primary care and the generalist approach in providing care to patients with complex, trauma-related physical and mental health issues.

Secondly, the heavy emotional load that primary care practitioners wear in relation to trauma-related work must be addressed. Understanding how practitioners can be supported as they navigate the complexity of their work is essential not only to promote clinician wellbeing and prevent burnout, but for the sustainability of the primary care workforce and long-term implementation of trauma-informed primary healthcare. Further research into how healthcare systems and services can be structured in a way that supports the wellbeing of primary care clinicians is needed.

Finally, further research, including further studies exploring practitioner and patient perspectives and evaluations of trauma-informed approaches, should continue to build an in-depth understanding of what high quality, evidence-based, trauma-informed primary healthcare looks like.

## Data Availability

All data and materials used during this systematic review are available from the corresponding author upon request.

## Declarations

### Registration and protocol

This review was not registered. A formal review protocol was not prepared.

## Abbreviations

ACES: Adverse childhood experiences
GP: General practitioner
SAMHSA: Substance Abuse and Mental Health Administration
CASP: Critical Appraisal Skills Programme
